# Hydrothermal synthesis of 3D hollow porous Fe_3_O_4_ microspheres towards catalytic removal of organic pollutants

**DOI:** 10.1186/1556-276X-9-648

**Published:** 2014-11-30

**Authors:** Xiansong Wang, He Huang, Guoqing Li, Yi Liu, Jiale Huang, Da-Peng Yang

**Affiliations:** 1 Department of Plastic and Reconstructive Surgery, National Tissue Engineering Center of China, 9th People's Hospital, Shanghai Jiao Tong University School of Medicine, Shanghai 200240, People’s Republic of China; 2 College of Chemistry and Life Science, Quanzhou Normal University, Quanzhou 362000, People’s Republic of China; 3 Department of Chemical and Biochemical Engineering, College of Chemistry and Chemical Engineering, Xiamen University, Xiamen 361006, People’s Republic of China

**Keywords:** Hydrothermal synthesis, Fe_3_O_4_ microspheres, Porous, Enzyme mimetics, Organic pollutants

## Abstract

Three-dimensional hollow porous superparamagnetic Fe_3_O_4_ microspheres were synthesized via a facile hydrothermal process. A series of characterizations done with X-ray diffraction, Brunauer-Emmett-Teller method, Fourier transform infrared spectroscopy, scanning electron microscopy, and transmission electron microscopy indicated that the production of Fe_3_O_4_ microspheres possessed good monodispersity, uniform size distribution, hollow and porous structural characters, and strong superparamagnetic behavior. The obtained Fe_3_O_4_ microspheres have a diameter of ca. 300 nm, which is composed of many interconnected nanoparticles with a size of ca. 20 nm. The saturation magnetization is 80.6 emu·g^-1^. The as-prepared products had promising applications as novel catalysts to remove organic pollutants (methylene blue) from wastewater in the presence of H_2_O_2_ and ultrasound irradiation.

## Background

In recent years, porous metal oxides have attracted considerable attention due to their potential applications in the field of lithium-ion batteries [1], drug delivery carrier [2], catalysis (including enzyme mimetics) [3], sensors [4], separation [5], and magnetic resonance imaging [6]. Among the porous metal oxides, iron oxide (Fe_3_O_4_) has become a particularly intriguing research target due to its low cost, good biocompatibility, as well as outstanding stability in physiological conditions [7-10]. Despite the great effort that has been made towards the synthesis of porous Fe_3_O_4_ with various sizes and morphologies, it still remains a big challenge to develop controlled and efficient ways for the synthesis of porous Fe_3_O_4_ microspheres with uniform size and strong magnetic performances in a large scale [11-13]. The hydrothermal method is one of the widely used methods for preparing functional inorganic nanomaterials [14-16]. It has a series of advantages for the resulting products, such as good crystallinity, uniform sizes, and special morphology, followed by excellent properties. Up to now, the Fe_3_O_4_ nanomaterials with different sizes and morphologies have been extensively reported by using this method. However, few reports are associated with the synthesis of 3D hollow porous Fe_3_O_4_ microsphere structures with uniform sizes on a large scale. Herein, we report a facile hydrothermal method for the construction of 3D hollow porous Fe_3_O_4_ microspheres from nanoparticle building blocks. Synthesis systems based on ethylene glycol (EG) and cetyl-methyl-ammonium bromide (CTAB) have been extensively adopted for the preparation of inorganic nanomaterials. In the whole reaction, EG was used as a solvent and CTAB as a dispersant. The as-prepared Fe_3_O_4_ microspheres exhibited porous structure, large surface area, strong superparamagnetic characters, as well as peroxidase-like activity, which are used as efficient enzyme mimetics to degrade organic pollutants (methylene blue).

## Methods

### Synthesis and characterization of 3D hollow porous Fe_3_O_4_ microspheres

All chemicals were of analytical grade and used as received without further purification (purchased from Sinopharm Chemical Reagent Co., Ltd., Shanghai, China). Typically, FeCl_3_ (0.8 g) was added into a beaker containing 40 mL ethylene glycol to become a clean yellow-brown solution at room temperature. Then, NaAc (3.6 g) and CTAB were subsequently added. The mixture solution was stirred vigorously for 10 min and was transferred into a Teflon-lined autoclave and heated to 200°C for 24 h. After cooling down to room temperature, the resulting products were collected and washed with deionized water and ethanol three times. The washed products were dried at 60°C for 1 day.

X-ray diffraction (XRD) patterns of the samples were recorded using a Bruker AXS micro-diffractometer (D8 ADVANCE; Bruker AXS GmbH, Karlsruhe, Germany) with Cu-Kα radiation (*λ* = 1.5406 Å) from 10° to 80° at a scanning speed of 0.33° min^-1^. The surface chemical groups of Fe_3_O_4_ microspheres were recorded by Fourier transform infrared spectroscopy (FTIR; Bruker Vector-22 FTIR spectrometer). The pore sizes and distribution curves were derived from the adsorption isotherm by employing the Barrett-Joyner-Halenda (BJH) method, and the surface areas were calculated through the Brunauer-Emmett-Teller (BET) equation. The magnetization versus magnetic field curves were measured at 300 K by a vibrating sample magnetometer (VSM; PPMS-9 T (EC-II), Quantum Design, San Diego, CA, USA). The surface morphology and structure were observed using a field emission scanning electron microscope (FESEM; Carl Zeiss AG, Oberkochen, Germany) operated at an accelerating voltage of 5.0 kV and a transmission electron microscope (TEM; JEM-2010, JEOL, Tokyo, Japan).

### Catalytic degradation of methylene blue

Methylene blue was employed as a model dye pollutant to evaluate the catalytic activity of 3D porous Fe_3_O_4_ microspheres for the activation of H_2_O_2_ under ultrasonic irradiation. Briefly, 0.5 mL of Fe_3_O_4_ microsphere stock solution (with different concentrations) was added into 10 mL aqueous solution of methylene blue (2 μg/mL) at pH 5.0. The mixed solution was put for 10 min to achieve adsorption-desorption equilibrium. Then, the degradation was done by rapidly adding H_2_O_2_ (with different concentrations) and was carried out with ultrasonic irradiation for 3 min. The solution was reacted for 20 min. Lastly, the Fe_3_O_4_ microspheres were collected using a magnet. The concentration of methylene blue in the clear solution was determined by measuring the absorbance of the solution at 662 nm on a UV-vis spectrophotometer (UV-2450, Shimadzu Co., Kyoto, Japan).

## Results and discussion

**Figure 1:**
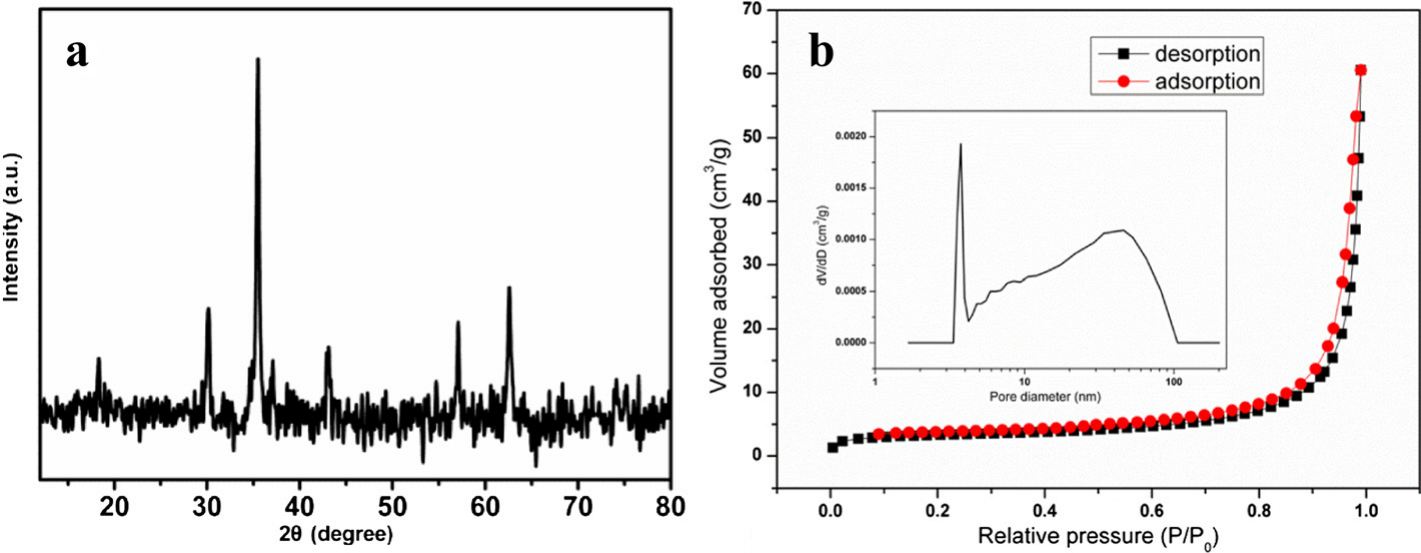
**XRD pattern (a) and N**_**2 **_**adsorption-desorption isotherms (b) of the as-prepared 3D hollow porous Fe**_**3**_**O**_**4 **_**microspheres.** The inset (in b) is the pore size distribution curve.

The chemical composition and phase purity of the resulting product was determined by XRD. As shown in Figure 1a, all the diffraction peaks can be assigned to the Fe_3_O_4_ phase (JCPDS card no. 65-3107). No other impurities are observed. The strongest diffraction peak (311) was used to estimate the crystalline size. According to the Debye-Scherrer formula, the calculated crystalline size is about 23 nm, which is consistent with the average grain sizes as observed in the following SEM images. The BET method of nitrogen adsorption/desorption was further employed to reveal the surface area and pore distribution of the as-obtained Fe_3_O_4_ products. The N_2_ adsorption-desorption isotherm and pore size distribution curve of the sample are shown in Figure 1b. The sample is a typical IV isotherm with a type-H3 hysteresis; there are two-level broad pore size distributions in the range of 3 to 5 nm and 40 to 100 nm (inset). In addition, the specific surface area of the Fe_3_O_4_ sample calculated by BET method is 148 m^2^ g^-1^. Such high surface area might be due to the existence of a hollow internal structure, which is confirmed by subsequent TEM observation.

**Figure 2:**
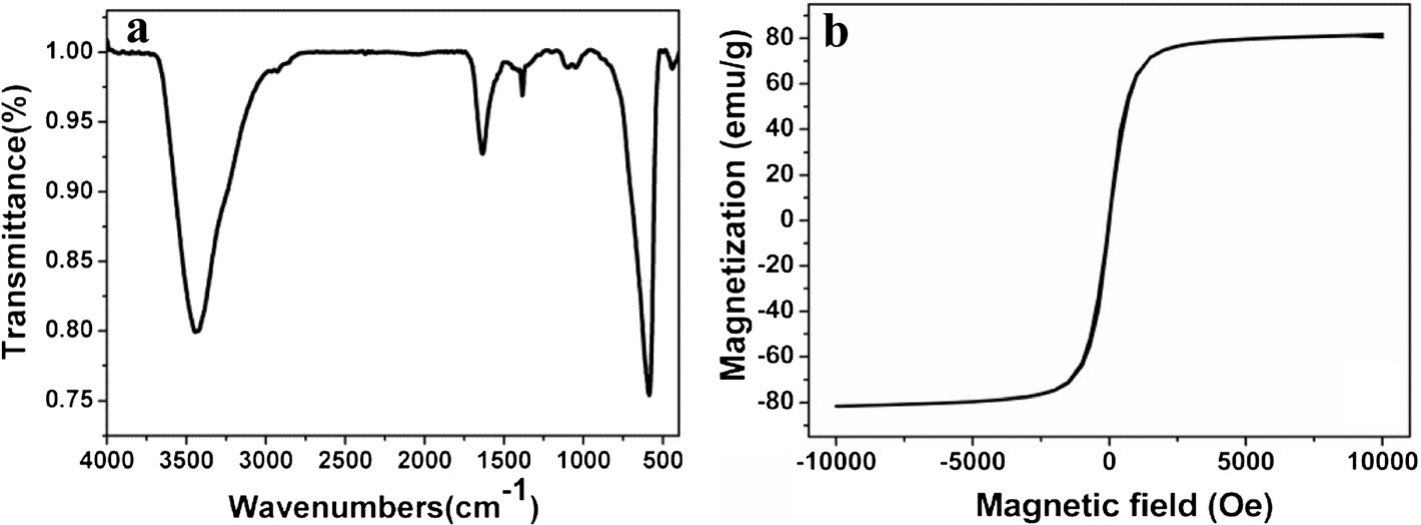
**FTIR spectrum (a) and magnetization curve (b) of the as-prepared 3D hollow porous F**_**3**_**O**_**4 **_**microspheres.**

The morphology of the sample was subsequently examined using both SEM and TEM. From Figure 2a, one can see that the as-obtained products were spherical in morphology, and the particles are highly dispersed. The average size of these particles is ca. 300 nm. From the SEM image with the higher magnification in Figure 2b, the surface morphology was clearly revealed. The individual Fe_3_O_4_ microsphere is composed of lots of interconnected nanoparticles with a size of ca. 20 nm. Many void spaces could be observed between nanoparticles, making the whole microsphere a porous structure. From a broken microsphere, it could also be clearly seen that the microsphere is hollow and composed of densely packed subunits. The hollow interior is further confirmed by TEM images. Figure 2c is the TEM image of Fe_3_O_4_ microspheres. The clear variation in contrast shows that the as-prepared F_3_O_4_ microsphere is hollow and has a porous structure. Meanwhile, the higher magnification of an individual microsphere (Figure 2d) indicated that a great number of particles connected to each other to form a 3D hollow porous structure, which is consistent with the SEM observation.

**Figure 3:**
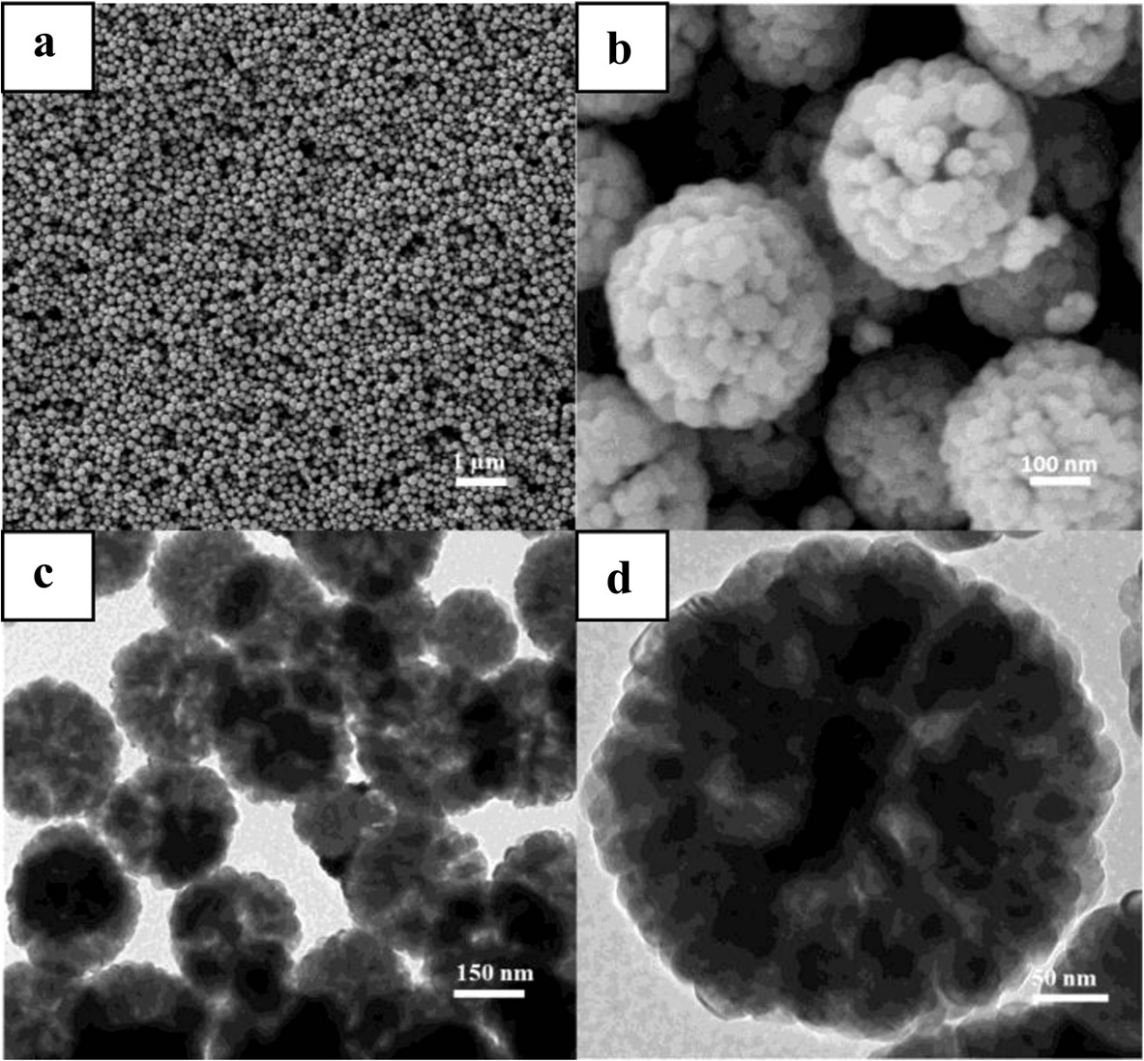
**SEM and TEM images of the as-prepared 3D hollow porous Fe**_**3**_**O**_**4 **_**microspheres.** SEM images: **(a)** low magnification and **(b)** high magnification. TEM images: **(c)** low magnification and **(d)** high magnification.

Figure 3a shows the magnetic curves of the prepared 3D hollow porous Fe_3_O_4_ microspheres. It does not show any hysteresis, revealing that the 3D hollow porous Fe_3_O_4_ microsphere has a typical superparamagnetic behavior. The saturation magnetization reaches up to 80.6 emu·g^-1^, which is higher than the PEG additive-synthesized Fe_3_O_4_ microspheres. The strong magnetic performances would be helpful to the removal of heavy metal ions or organic pollutant in waste water. To determine the surface chemical compositions, FTIR measurement was performed in the range of 400 to 4,000 cm^-1^. As shown in Figure 3b, the peak at 3,453 cm^-1^ was assigned to the -OH stretch. The peaks at 605 and 448 cm^-1^ can be assigned to the vibrations of Fe-O. Other characteristic peaks at 1,638, 1,402, and 1,045 cm^-1^ were due to the CTAB molecules. The results showed that the 3D hollow porous microspheres were coated by a layer of CTAB molecules.

Based on the above results, a possible formation mechanism of the 3D hollow porous microspheres was put forward as follows: The ethylene glycol acts as both a solvent and a reducer during the solvothermal process. On one hand, it can afford -OH groups to coordinate with Fe^3+^. With the changes of pH (the addition of NaAc), high temperature, as well as high pressure, the Fe_3_O_4_ nuclei are generated; they quickly grow up to become small nanoparticles and aggregate to form the microspheres, owing to the high surface energy. With a longer reaction time, the microspheres continue to grow up and finally form the hollow porous structure, probably owing to the Ostwald ripening. The addition of CTAB is used to control and disperse the resulting products. In fact, the detailed mechanism is rather complex and remains a further discussion to materials chemists.

**Figure 4:**
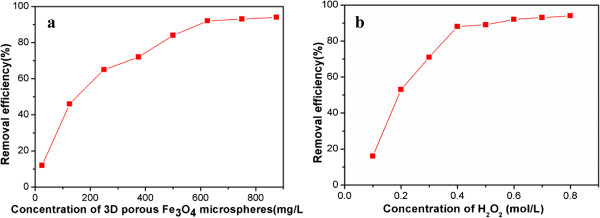
**Removal efficiency of methylene blue. (a)** Effect of the concentration of 3D hollow porous Fe_3_O_4_ microspheres on the removal efficiency of methylene blue (2 μg/mL) in the presence of H_2_O_2_ (0.4 M) and ultrasound irradiation, pH = 5.0. **(b)** Effect of the concentration of H_2_O_2_ on the removal efficiency of methylene blue (2 μg/mL) in the presence of 3D hollow porous Fe_3_O_4_ microspheres (2 μg/mL), pH = 5.0.

The treatment of organic dyes in wastewater is a challenging task in industries. Considering the unique structure and large surface area of Fe_3_O_4_ microspheres, it is expected that a strong affinity to organic molecules can be achieved by using these Fe_3_O_4_ microspheres as sorbent material [17-21]. Furthermore, a recent research has shown that Fe_3_O_4_ magnetic nanoparticles have a peroxidase-like activity [3,17]. In the presence of H_2_O_2_, Fe_3_O_4_ nanoparticles could efficiently do catalytic oxidation of organic dyes via the well-known Fenton reaction [22]. To assess the catalytic ability of the 3D hollow porous Fe_3_O_4_ microspheres to the activation of H_2_O_2_, herein, methylene blue is chosen as the model compound of organic pollutants, which is a widely used staining agent. Figure 4a shows the influence of 3D hollow porous Fe_3_O_4_ concentrations on the degradation rates of methylene blue in the presence of H_2_O_2_ (certain concentration). One can see that the removal efficiency of methylene blue increases followed by the increase of Fe_3_O_4_ concentrations (reaction time is 30 min). When the concentration of Fe_3_O_4_ reaches up to 600 mg/L, the removal efficiency of organic dyes approaches 90%. With the continuous rise of the concentration of Fe_3_O_4_ microspheres, the removal efficiency has no obvious changes, indicating that a saturation state is completed. Therefore, 600 mg/mL is applied as the optimal concentration for methylene blue clearance. On the other hand, the concentration of H_2_O_2_ has also influenced the removal efficiency of methylene blue. In the absence of H_2_O_2_, methylene blue is partly removed (about 10%), indicating that 3D hollow porous Fe_3_O_4_ microspheres can adsorb organic pollutants. In the presence of H_2_O_2_ with different concentrations, the removal efficiency was obviously enhanced, as shown in Figure 4b. When the concentration of H_2_O_2_ is 0.6 mol/L, the removal efficiency of methylene blue reaches the maximum (near 90%). Accordingly, an optimal concentration of H_2_O_2_ at 0.6 mol/L was recommended for practical use.

Based on the above results, a possible catalytic mechanism of 3D hollow porous Fe_3_O_4_ as the peroxidase mimetic is proposed as follows: due to the large surface and unique structures, H_2_O_2_ and methylene blue molecules can be adsorbed onto the surface of 3D hollow porous Fe_3_O_4_ microspheres. Under the influence of ultrasound irradiation, H_2_O_2_ molecules are activated by the bound Fe^2+^ and Fe^3+^ of Fe_3_O_4_ microspheres to generate reactive oxygen species (including · OH, O_2_^-^˙, HO_2_˙). These radicals can further attack organic pollutants to degrade them.

## Conclusions

In summary, we have developed a simple one-pot hydrothermal procedure for the synthesis of 3D hollow porous Fe_3_O_4_ microspheres composed of lots of nanoparticles assembled on a large scale. The as-prepared microspheres have good dispersibility, large BET surface, strong superparamagnetic performance, as well as peroxidase-like activity, which can be used as a kind of adsorbent and catalytic materials for the removal of organic pollutants. We believe that the easy preparation method, the large-scale output, the unique structure, as well as the outstanding performances endow these 3D microspheres many promising applications ranging from biomedicine to energy materials as well as environmental remediation.

## Competing interests

The authors declare that they have no competing interests.

## Authors’ contributions

XW and JH conceived the study and designed the experiments. XW and YL performed the experiments. XW, YL, GL, and HH analyzed the data. JH and DPY contributed the materials and analysis tools. DPY wrote the manuscript. All authors read and approved the final manuscript.
